# Assessing the treatment of male infertility through principal component analysis: a retrospective study

**DOI:** 10.1186/s12610-026-00317-1

**Published:** 2026-07-07

**Authors:** Muhammed Ali Kamal, Kawa M. Jamal Rashid

**Affiliations:** 1 College of Basic Education, University of Sulaimani, Sulaymaniyah, Kurdistan Region Iraq; 2 College of Administration and Economics, University of Sulaimani, Sulaymaniyah, Kurdistan Region Iraq

**Keywords:** Infertility, Male, Semen, Sperm Motility, Sperm Morphology, Principal Component Analysis, Infertilité, Homme, Sperme, Mobilité des Spermatozoïdes, Morphologie des Spermatozoïdes, Analyse des Composantes principales

## Abstract

**Background:**

To determine whether routine fertility treatments reorganize the systemic architecture of semen parameters in infertile men, rather than producing isolated parameter improvements. We specifically investigated whether morphology becomes more functionally integrated with motility and other core fertility indicators following treatment.

**Materials and methods:**

Paired pre and posttreatment semen samples from 112 infertile men attending Faruk Medical City, Kurdistan Region of Iraq (2015–2024), were analyzed. Data included age, liquification time, abstinence days, white blood cells, semen volume, sperm concentration, total sperm number, progressive motility, normal morphology, and sperm defects. Non-normal data were transformed, and Spearman’s correlations and Principal Component Analysis (PCA) were used to characterize systemic relationships before and after treatment.

**Results:**

Semen parameter relationships showed reorganization following treatment, rather than isolated gains. Core indicators (concentration, sperm number, motility) maintained structural stability (Principal Component (PC)1 variance: 34%→35%), while morphology became more integrated with other parameters. Correlations between morphology and concentration increased 51% (rs = 0.327→0.494) and with motility 32% (rs = 0.453→0.597). PCA confirmed this reorganization, with PC2 transitioning from a ‘structural defect’ component toward a pattern consistent with a ‘functional integration’ axis coupling morphology and motility. Age-stratified analysis revealed stronger morphology–motility coordination in younger men, whereas older men showed persistent viscosity and defect issues. Leukocyte–defect correlations weakened, a pattern consistent with reduced inflammatory contributions to sperm abnormalities.

**Conclusions:**

Fertility treatments were associated with systemic reorganization of semen parameter networks, with morphology emerging as a central biomarker of functional recovery. These findings support a shift from single-parameter monitoring toward multivariate, systems-based approaches and highlight the value of age-stratified treatment strategies and anti-inflammatory interventions in optimizing outcomes.

**Supplementary Information:**

The online version contains supplementary material available at 10.1186/s12610-026-00317-1.

## Résumé

**Contexte:**

Déterminer si les traitements de routine à visée de fertilité réorganisent l’architecture systémique des paramètres du sperme chez les hommes infertiles, plutôt que de produire des améliorations isolées des paramètres. Nous avons spécifiquement étudié si la morphologie s’intègre davantage fonctionnellement avec la mobilité et d’autres indicateurs clés de fertilité après le traitement. Pour cela, nous avons analysé des échantillons de sperme appariés avant et après traitement de 112 hommes infertiles ayant fréquenté Faruk Medical City, région du Kurdistan en Irak (2015–2024). Les données comprenaient l’âge, le temps de liquéfaction, le délai d’abstinence, les globules blancs, le volume de sperme, la concentration de spermatozoïdes, le nombre total de spermatozoïdes, la mobilité progressive, la morphologie normale et les anomalies des spermatozoïdes. Les données non normales ont été transformées, et les corrélations de Spearman ainsi que l’analyse des composantes principales (ACP) ont été utilisées pour caractériser les relations systémiques avant et après le traitement.

**Résultats:**

Les relations entre les paramètres du sperme ont montré une réorganisation après traitement, plutôt que des gains isolés. Les indicateurs de base (concentration, nombre de spermatozoïdes, mobilité) maintenaient une stabilité structurelle (variance des composantes principales (CP)1 : 34 %→35 %), tandis que la morphologie s’intégrait davantage aux autres paramètres. Les corrélations entre la morphologie et la concentration ont augmenté de 51 % (rs=0,327→0,494) et avec la mobilité de 32 % (rs=0,453→0,597). L’analyse des composantes principales (ACP) a confirmé cette réorganisation, CP2 passant d’un composant de « défaut structurel » à un motif cohérent avec un axe « d’intégration fonctionnelle » couplant morphologie et mobilité. L’analyse stratifiée par âge a révélé une meilleure coordination morphologie-mobilité chez les hommes plus jeunes, tandis que les hommes plus âgés présentaient des problèmes persistants de viscosité et de d’anomalies. Les corrélations leucocyte-anomalie se sont affaiblies, un schéma cohérent avec une diminution des contributions inflammatoires aux anomalies des spermatozoïdes.

**Conclusions:**

Les traitements de fertilité étaient associés à une réorganisation systémique des réseaux de paramètres du sperme, la morphologie émergeant comme un biomarqueur central de la récupération fonctionnelle. Ces résultats soutiennent un passage de la surveillance à paramètre unique à des approches multivariées basées sur des systèmes, et soulignent la valeur des stratégies de traitement stratifiées par âge ainsi que des interventions anti-inflammatoires pour optimiser les résultats.

## Introduction

Male infertility accounts for nearly half of all infertility cases and affects approximately 15% of couples worldwide [1]. Fertility potential in man is based on sperm parameters and while seminal analysis which underpins male fertility assessment is considered to be very important, it has been historically interpreted using isolated variables such as concentration, motility and morphology [2]. However, male fertility is a multivariate biological process where the interrelationships between these parameters may be more informative than their absolute values. Treating them as isolated metrics risks overlooking systemic dysfunction. One of the key aspects of this systems-level view is that semen parameters are not to be considered as individual entities [3].

Such systems-level insights are of particular significance given that fertility treatments generally lead to simultaneous changes across these multiple variables. Varicocele repair, hormonal therapy, and antioxidants can together influence concentration, motility, and morphology [1], yet their network-level effects remain largely unexplored. It could also show us whether patterns consistent with a natural coordinated morphology–motility relationship are observed following successful interventions, rather than improvement on a single isolated metric. This insight may support a shift in treatment monitoring from tracking individual parameters toward evaluating multivariate coherence across the semen profile. This gap is clinically significant because fertility may depend not just on optimizing single metrics but on reestablishing coordinated sperm function. Elucidating system-level reorganization would help uncover new biomarkers for treatment response, predict more responsive phenotypes to specific treatments and understand the mechanism of action of a treatment.

Kurdistan Region of Iraq offers a relevant population for studying systemic treatment response, given documented differences in baseline semen profiles compared to Western populations [4] and dietary patterns associated with better semen quality [5]. These characteristics suggest that treatment response patterns may differ across populations, making regional studies valuable for understanding whether systems-level reorganization follows universal biological principles. In local studies semen abnormalities have been reported to be highly prevalent among infertile males [6, 7], with age, obesity, and smoking consistently associated with abnormal parameters [8, 9], underscoring the importance of examining treatment responses stratified by demographic factors. Retrospective national studies have similarly also documented declining sperm concentration and strong correlations among seminal parameters, these results also argue for the use of multivariate techniques [10].

Principal Component Analysis (PCA) offers a robust tool for uncovering hidden patterns in multivariate datasets. By reducing dimensionality, PCA represents shared variance between parameters, detects latent variables like “overall semen quality,” and limits multicollinearity. PCA has been previously applied in developing composite semen quality scores, which were demonstrated to better correlate with fertility outcomes than individual parameters [11], and for diagnosis of sperm health status using automated classification methods [12], as well as in differentiating between semen specimens by integration of metabolic data together with sperm related parameters to have a deep view on the fertility assessment [13]. However, to our knowledge, no study has systematically examined pre- and posttreatment changes in semen parameter networks using PCA.

We hypothesized that fertility treatment success involves systematic reorganization of semen parameter networks rather than isolated improvements in individual metrics. Given that quality of semen decreases with age, we also investigated whether treatment-related reorganization differed across ages and expected to observe more pronounced parameter improvements in younger males. Using non-parametric correlation analysis and PCA, we aimed to (i) clarify the latent factor structure in semen parameters pre and posttreatment, (ii) characterize the longitudinal changes between pre and posttreatment assessments, and (ii) earmark new emerging patterns of parameter integration that could serve as biomarkers for clinical decision-making. This systems-biology framework provides foundation for precision fertility medicine and may direct personalized treatment interventions in this and similar populations.

## Materials and methods

### Patients

This observational, longitudinal, retrospective effectiveness study analyzed anthropometric data and semen parameters from men attending the In Vitro Fertilization (IVF) center at Faruk Medical City (FMC), Sulaymaniyah, Kurdistan Region of Iraq. Two visits per patient between April 2015 and May 2024 were considered, yielding 224 samples from 112 infertile men (see Supplementary Table 1 for patient characteristics). Semen analysis results from laboratory and other data are anonymously collected and retrieved from their medical records. Patients with azoospermia were excluded. Patients underwent routine clinical management as determined by their treating physicians. Treatment modalities were heterogeneous and were not stratified in the present analysis.

### Treatment protocol

Individualized treatment protocols were applied based on the fertility diagnosis of each patient (see Supplementary Fig. 1 for the treatment protocol). Due to the retrospective design, detailed treatment regimens were not systematically documented. The median interval between pre and posttreatment semen analyses was 163 days (~ 5.4 months), aligning with one full spermatogenesis cycle and sufficient time for therapeutic effects to manifest. Abstinence periods were standardized to 2–7 days (median: 3 days) before both analyses to achieve comparability.

### Statistical analysis

Statistical analyses were performed using IBM SPSS Statistics, Version 24.0. Shapiro-Wilk test is used to test if data in different variables is normally distributed. Spearman’s rank correlation is used to explore the relationship between the variables. To mitigate non-normality and outliers prior to PCA, logarithmic transformations were applied to right-skewed variables with extreme outliers (liquification time, WBC, abstinence days, volume, sperm concentration, total sperm number, normal morphology, tail defects posttreatment); square-root transformations were applied to moderately skewed variables (sperm concentration and total sperm number pretreatment); and winsorization at the 5th and 95th percentiles was applied to variables where transformation alone did not sufficiently reduce the influence of extreme values (normal morphology and WBC posttreatment). These procedures were applied to PCA input data only; Spearman’s correlations were conducted on untransformed ranked data and required no transformation.

Transformation methods were selected based on the severity and direction of skewness identified in the Shapiro-Wilk normality assessment, with the goal of approximating symmetrical distributions suitable for PCA input while preserving the relative structure of the data.

Variable selection for each PCA model followed a systematic iterative procedure. Twelve clinically reasonable variable subsets were evaluated for each timepoint. The best model was selected according to four criteria tested together: (i) highest overall achievable Kaiser–Meyer-Olkin measures (KMO) [14], (ii) maximum explained variance, (iii) the simplicity of component structure and (iv) unconditional retention of core WHO semen parameters (concentration, motility, morphology). The determinant of the correlation matrix was computed for all combinations to confirm that no singularity due to multicollinearity and all values were far above the threshold of 0.00001.

Pretreatment PCA excluded four variables: Volume (X_5_, individual MSA = 0.222), Neck Defects (X_11_, severe multicollinearity with Head Defects, *r* = − 0.820), Abstinence Days (X_3_, procedural variable standardized across patients with minimal contribution to biological variance), and Age (X_1_, weak associations with semen parameters pretreatment). This combination achieved the highest KMO across all tested subsets (0.655) with three components explaining 71% of variance.

Posttreatment PCA excluded five variables: Volume (X_5_, MSA = 0.285), Abstinence Days (X_3_, consistently poor factorial relevance across both timepoints), WBC (X_4_, MSA = 0.468, falling below the acceptable threshold posttreatment), Head Defects (X_10_, severe multicollinearity with Neck Defects, *r* = − 0.824), and Tail Defects (X_12_, MSA = 0.133). This combination achieved the highest KMO across all tested posttreatment subsets (0.610) with three components explaining 69% of variance. KMO values of 0.61–0.66 are considered acceptable for biological data where multiple independent physiological processes contribute to observed variance, and Bartlett’s tests confirmed adequate correlation structure for PCA at both timepoints (pretreatment: χ² = 373.7, df = 28, *p* < 0.001; posttreatment: χ² = 238.2, df = 21, *p* < 0.001). Data visualization, specifically heatmap creation, was performed using Python 3.12.3 in Visual Studio Code within the Anaconda environment.

### Ethical considerations

This study was conducted in accordance with the Declaration of Helsinki. Due to the retrospective nature of the study and the use of anonymized data, the requirement for informed consent was waived. The study protocol was reviewed and approved by Scientific committee at the College of Administration and Economics (No 35/9 on 06.04.2025) at the University of Sulaimani. Data collection was approved by the FMC Research and Ethics Committee to be anonymous.

## Results

Shapiro–Wilk tests were performed on the 12 study variables pre- and posttreatment. Except for age, all variables significantly deviated from normality (*p* < 0.05), supporting the use of non-parametric analyses (Table 1).

**Table 1:** Normality of variables before and after treatment using the Shapiro–Wilk test (α = 0.05)

Variable		Pretreatment			Posttreatment		
	Sample Size (*n*)	W	*p*	Normality	W	*p*	Normality
Age (X_1_)	112	0.981	0.106	Normal	0.979	0.068	Normal
Liquification Time (X_2_)	112	0.459	< 0.001	Non-normal	0.463	< 0.001	Non-normal
Abstinence Days (X_3_)	112	0.625	< 0.001	Non-normal	0.787	< 0.001	Non-normal
WBC (X_4_)	112	0.500	< 0.001	Non-normal	0.488	< 0.001	Non-normal
Volume (X_5_)	112	0.915	< 0.001	Non-normal	0.909	< 0.001	Non-normal
Sperm Concentration (X_6_)	112	0.909	< 0.001	Non-normal	0.897	< 0.001	Non-normal
Total Sperm Number (X_7_)	112	0.834	< 0.001	Non-normal	0.778	< 0.001	Non-normal
Progressive Motility (X_8_)	112	0.934	< 0.001	Non-normal	0.931	< 0.001	Non-normal
Normal Morphology (X_9_)	112	0.469	< 0.001	Non-normal	0.323	< 0.001	Non-normal
Head Defects (X_10_)	112	0.932	< 0.001	Non-normal	0.956	0.001	Non-normal
Neck Defects (X_11_)	112	0.924	< 0.001	Non-normal	0.940	< 0.001	Non-normal
Tail Defects (X_12_)	112	0.873	< 0.001	Non-normal	0.811	< 0.001	Non-normal

Shapiro–Wilk test (α = 0.05) is used.W = Shapiro–Wilk statistic, *WBC* White blood cells. Variables with *p* < 0.05 were considered non-normally distributed

**Fig. 1:**
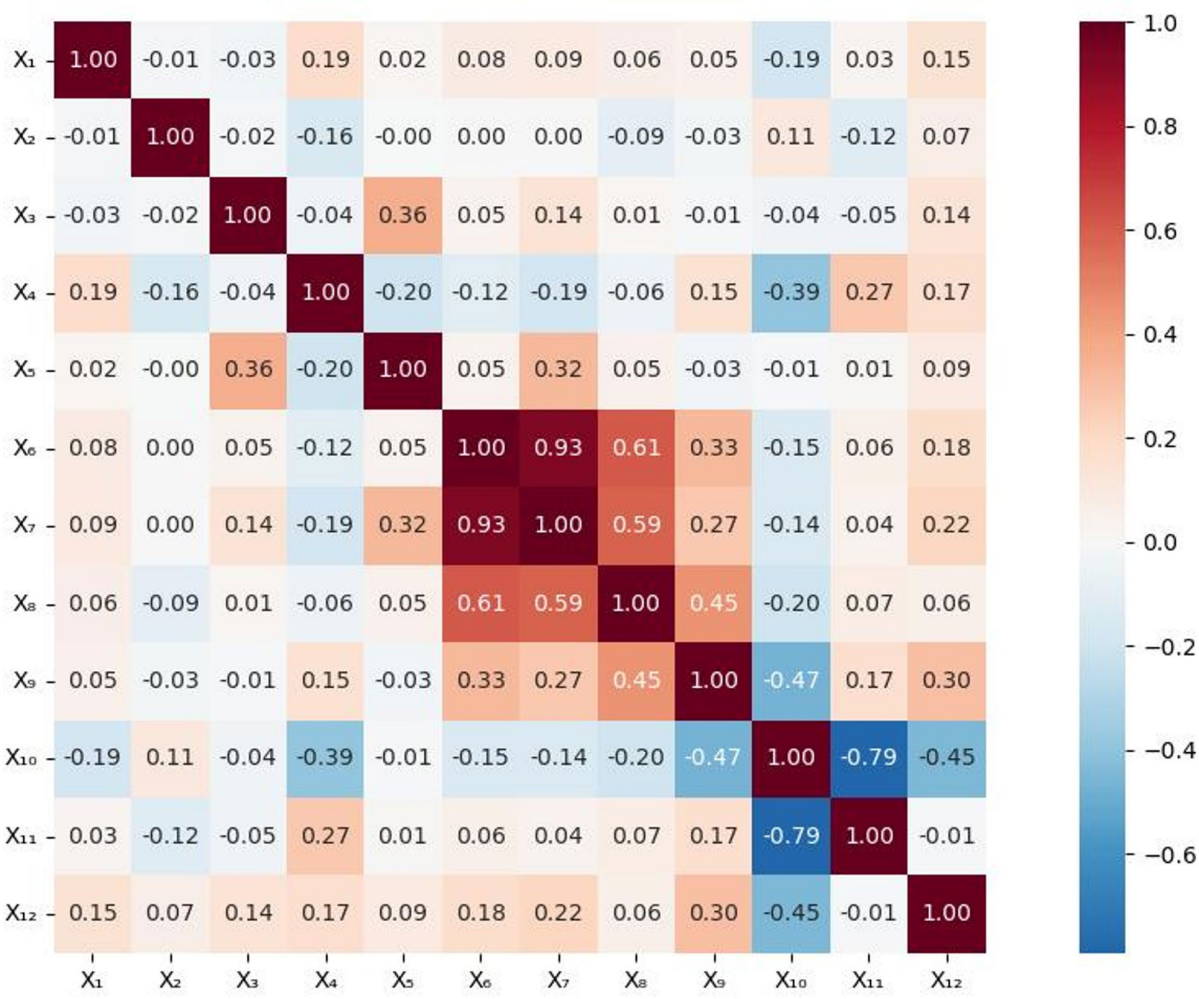
Spearman’s correlation heatmap of the pretreatment variables. Spearman’s correlation heatmap of pretreatment variables. Blue shades indicate negative correlations, brown shades indicate positive correlations (darker color means stronger)

Pretreatment correlations (Fig. 1) uncovered two dominant clusters: parameters related to quantity (sperm concentration (X_6_), total sperm number (X_7_), progressive motility (X_8_)) and associations between morphology (X_9_) with shape abnormalities (normal morphology, head (X_10_), neck (X_11_), tail defects (X_12_)). Sperm concentration (X_6_) strongly correlated with total sperm number (X_7_), (Spearman’s Rank Correlation Coefficient (*rs*) = 0.93)) and motility (X_8_), (*rs* = 0.61), and weakly with morphology (X_9_), (*rs* = 0.33). Total sperm number (X_7_) and motility (X_8_) were also moderately associated with morphology (X_9_). Among defects, head defects (X_10_) negatively correlated with neck (X_11_) (*rs* = − 0.79) and tail defects (X_12_) (*rs* = − 0.45). White Blood Cell (WBC) counts (X_4_) showed weak negative associations with semen quality, while age (X_1_) correlated weakly with WBC (X_4_) and head defects (X_10_).

**Fig. 2:**
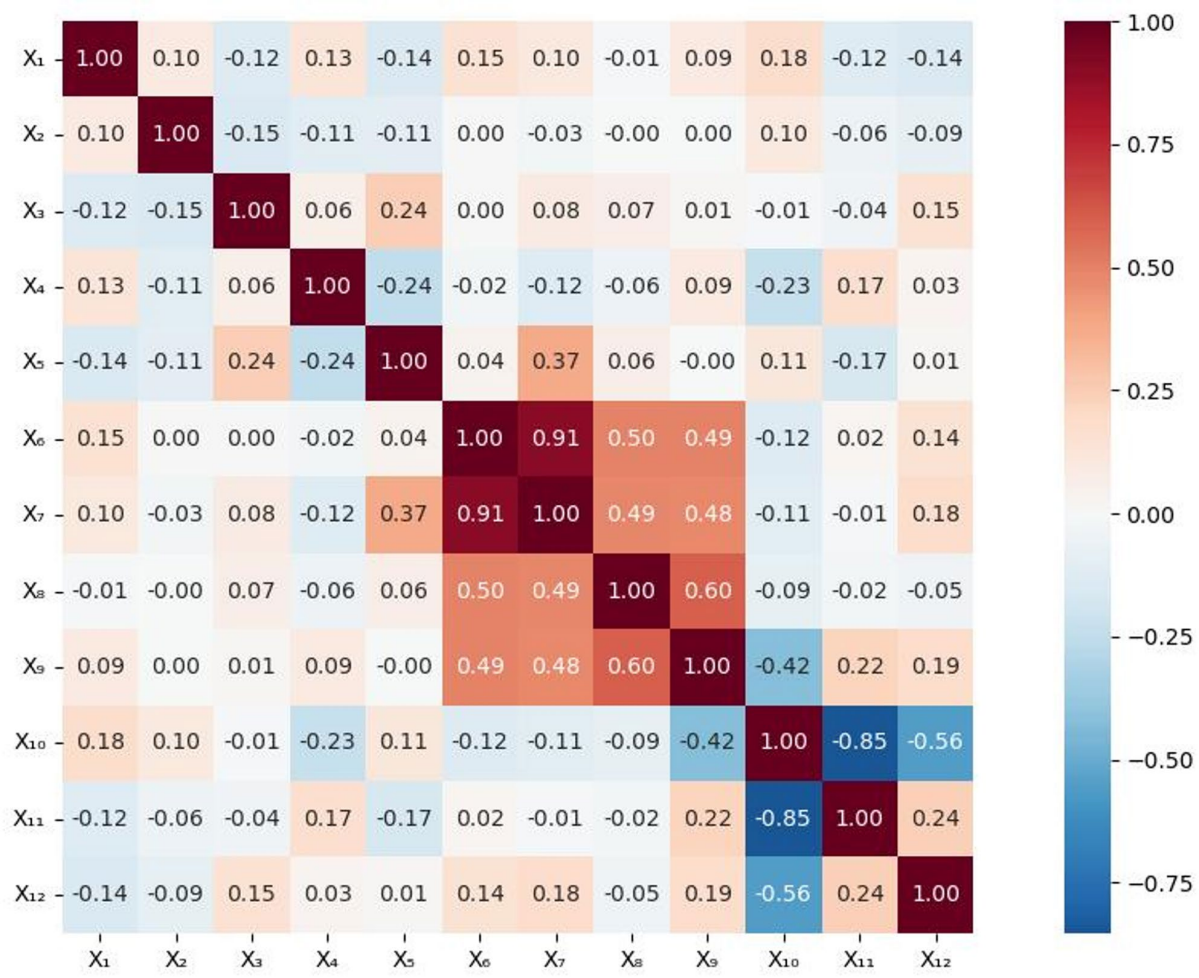
Spearman’s correlation heatmap of the posttreatment variables. Spearman’s correlation heatmap of posttreatment variables. Blue shades indicate negative correlations, brown shades indicate positive correlations (darker color means stronger)

**Fig. 3:**
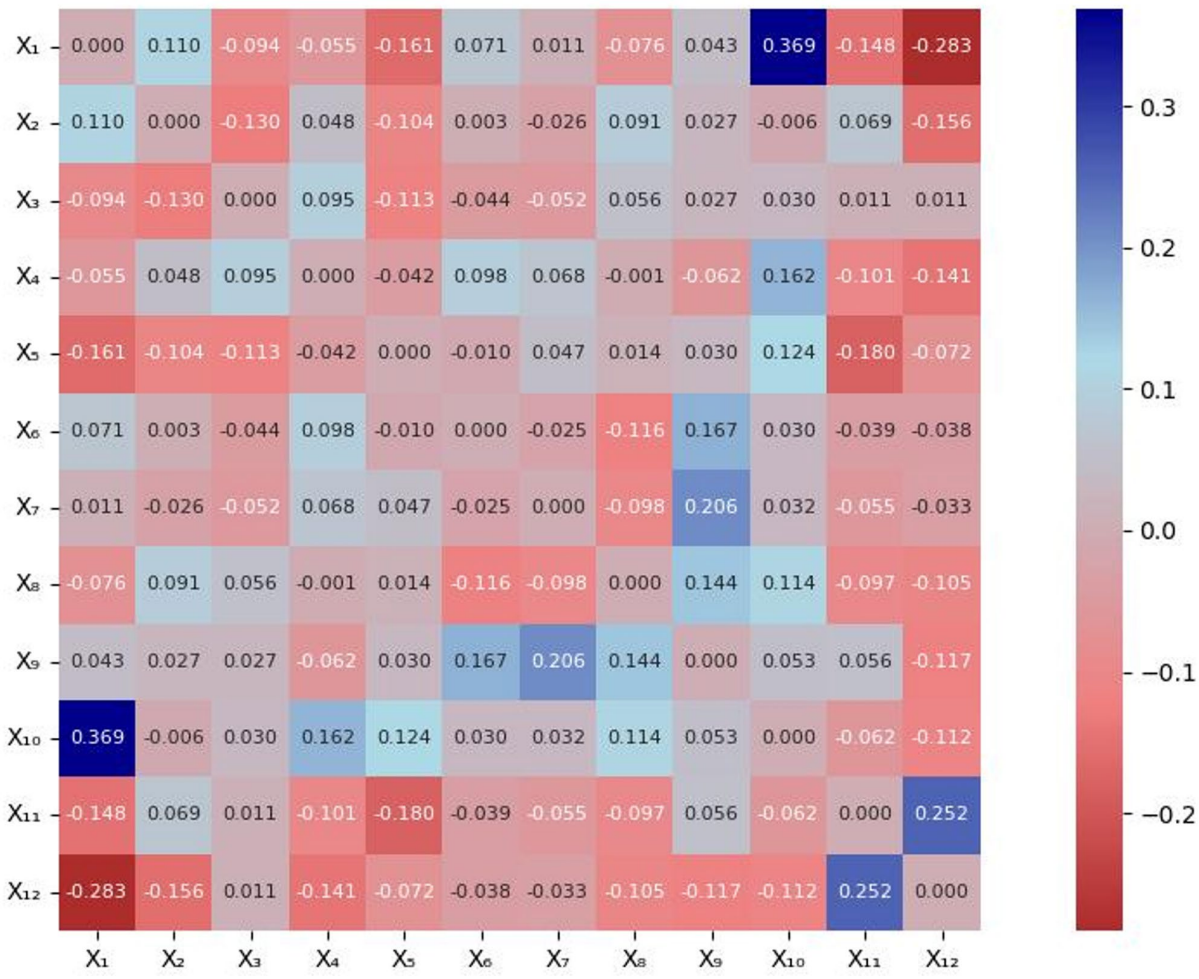
Spearman’s correlation differences pre and posttreatment. Delta heatmap of the difference between pretreatment and posttreatment variables, with blue indicating strengthened correlations and red weakened ones

Posttreatment correlations (Fig. 2) retained these clusters with strengthened morphology–quantity relationships. Sperm concentration (X_6_) and total sperm number (X_7_) remained strongly correlated (*rs* = 0.91), while associations with motility (X_8_) and morphology (X_9_) increased. Normal morphology (X_9_) correlated more positively with motility (X_8_) and moderately negatively with head defects (X_10_). Delta heatmap (Fig. 3) highlights these shifts between pre and posttreatment correlations.

Among pretreatment correlations, sperm concentration (X_6_) and total sperm number (X_7_) showed the strongest association (*rs* = 0.93, *p* < 0.001), while normal morphology (X_9_) showed moderate correlations with motility (X_8_) (*rs* = 0.45) and weaker correlations with total sperm number (X_7_) (*rs* = 0.27).

Posttreatment, these relationships were strengthened: normal morphology (X_9_) correlations increased by 32–76% with motility (X_8_), sperm concentration (X_6_), and total sperm number (X_7_), as shown in Fig. 2.

PCA reflected these patterns. Pretreatment PCA identified PC1 dominated by sperm quantity and motility, PC2 by structural defects and morphology, and PC3 by liquification time and WBC count (Table 2). Posttreatment, PC1 again captured quantity, while PC2 mirrored integrated morphology–motility improvements, consistent with the strengthened correlations observed in the heatmaps (Figs. 1 and 2; Table 3).

**Table 2:** Rotated component matrix for pretreatment PCA

Variables	PC1	PC2	PC3
Liquification Time (X_2_)	-0.11	-0.04	**0.89**
WBC (X_4_)	-0.28	0.49	**-0.50**
Sperm Concentration (X_6_)	0.**94**	0.09	0.04
Total Sperm Number (X_7_)	**0.93**	0.08	0.10
Progressive Motility (X_8_)	**0.79**	0.10	-0.17
Normal Morphology (X_9_)	0.13	**0.66**	-0.17
Head Defects (X_10_)	-0.13	**-0.86**	0.20
Tail Defects (X_12_)	0.08	**0.76**	0.20

Loadings ≥ |0.50| are bolded. Statistical analysis: Principal Component Analysis (PCA); extraction method: PCA; rotation method: Varimax with Kaiser normalization

**Table 3:** Rotated component matrix for posttreatment PCA

Variables	PC1	PC2	PC3
Age (X_1_)	0.34	**-0.54**	0.45
Liquification Time (X_2_)	-0.03	-0.10	**0.73**
Sperm Concentration (X_6_)	**0.94**	0.10	-0.03
Total Sperm Number (X_7_)	**0.92**	0.09	-0.08
Progressive Motility (X_8_)	**0.58**	**0.59**	0.03
Normal Morphology (X_9_)	0.20	**0.81**	0.06
Neck Defects (X_11_)	0.08	-0.11	**-0.74**

Loadings ≥ |0.50| are bolded. Statistical analysis: Principal Component Analysis (PCA); extraction method: PCA; rotation method: Varimax with Kaiser normalization

These PCA components provide a dimensional summary of the interrelated semen parameter structure at each timepoint. The differing variable sets between pre- and posttreatment PCA models demonstrate the data-driven nature of the iterative optimization process: the correlation structure among parameters differed between timepoints, so that a distinct set met adequacy thresholds at each timepoint. Importantly, WBC made a meaningful contribution to the pretreatment model but lost factorial significance in the posttreatment model, a pattern explored further in the Discussion. Notably, both models retained the core WHO fertility parameters (concentration, motility, morphology), so that comparisons of the principal components describing intrinsic semen quality can be made meaningfully across timepoints. We note that this difference in the sets of variables is a limitation on direct structural equivalence between models, and thus results should be interpreted as independent characterizations of each timepoint instead of a longitudinal structural analysis fully matched across the timepoints.

## Discussions

The bivariate correlations and principal component analyses reveal complementary patterns that illuminate both the stability and plasticity of semen parameter relationships following treatment. The convergent evidence from these analytics implies some clinically relevant observations that merit further exploration. The pretreatment correlation matrix showed the anticipated strong interrelations of sperm concentration, total sperm number and progressive motility (rs ≥ 0.590, *p* < 0.001), which persisted posttreatment albeit with some attenuation. This stability is mirrored in PCA results, with these three parameters consistently driving PC1 during both analyses and explaining 34% and 35% of variance pretreatment and posttreatment, respectively. Instead, the decreased loading of progressive motility in PC1 (0.79 → 0.58) coincides with its emergence as a strong contributor in posttreatment PC2 (loading: 0.59), suggesting a pattern consistent with functional reorganization, as reflected in longitudinal changes between pre and posttreatment assessments, where motility showed dual associations with both production capacity and morphological quality. The clinical meaning of this finding suggests that successful treatment may be associated with multidimensional rather than isolated changes across semen parameters. Our PCA results demonstrate that the relationship between strengthened parameters is not isolated to one parameter; it displays associations with treatment, supported by longitudinal changes between pre and posttreatment assessments, as described in WHO guidelines which highlight these parameters as interdependent [2]. The most surprising result comes from the strong increases in morphology-related correlations observed posttreatment.

Normal morphology correlations with sperm concentration increased 51% (0.327 → 0.494), with progressive motility by 32% (0.453 → 0.597), and with total sperm number by 76% (0.270 → 0.476). These correlation changes are substantiated by the PCA structural reorganization, where posttreatment PC2 reconfigured from a “structural defect” component (dominated by head and tail defects) to a “coordinated quality” component combining morphology (loading: 0.81) and motility (loading: 0.59). This coordinated restructuring is consistent with concurrent changes in sperm form and function observed in a pre– and posttreatment comparison, suggesting a shift beyond isolated parameter changes toward greater overall functional coherence — albeit prospective experimental validation for this interpretation remains, of course, necessary to substantiate. Abnormalities in sperm morphology are often associated with lesser motility and so this interpretation is informed by evidence for the functional interdependence of these two aspects of fertilization success [15]. The findings raise the hypothesis that more effective treatments may be associated with a partial normalization of such natural associations, potentially explaining why morphologically normal sperm show superior motility patterns after treatment — a hypothesis which should be tested directly in prospective studies with clinical outcome data. The age-associated patterns detected in posttreatment analyses lead to an especially novel finding. Age was not included in pretreatment PCA because it weakly correlated with other parameters, but it did emerge as a significant posttreatment factor loading negatively on the morphology-motility component (-0.544) and positively on the liquification-neck defect component (0.445). This pattern is supported by the correlational analysis, where age had stronger correlations with treatment outcomes in younger patients.

The PCA evidence is consistent with younger patients experiencing more pronounced changes in the morphology-motility axis, while older patients appear to show persistent challenges with semen viscosity and structural defects — though these age-stratified patterns should be interpreted cautiously given the retrospective design and absence of stratified subgroup analyses. The age-stratified response pattern recognized here has significant clinical implications for treatment counseling that may guide age-adjusted therapeutic modalities. Previous publications have documented the age-related decline in sperm quality [16, 17] but our results uniquely show how patterns of treatment responsiveness change with advancing male age.

The weakening of white blood cell associations with morphological defects (head defects: -0.387 → -0.225) parallels the exclusion of WBC from posttreatment PCA, consistent with a reduction in inflammatory contributions to sperm abnormalities following treatment. Similarly, the attenuation of the correlation between progressive motility and head defects (-0.203 → non-significant) is consistent with a weakening of the association between structural abnormalities and functional impairment following treatment, though this remains an observational finding requiring prospective confirmation. The maintenance of strongly negative correlations between different defect categories in both correlation and PCA analyses (head-neck defects: -0.791 → -0.853) indicates that although total defects burden may be less, the underlying complementary relationship of structural abnormalities within sperm seems to remain unchanged.

This high-resolution analysis lays the groundwork for targeted therapeutic strategies that would intervene on patterns of specific morphological defects rather than relying on global enhancer strategies. The combination of correlation and PCA results highlights some clinically actionable findings:

1. The emergence of the posttreatment component (PC2) and the concurrent strengthening of correlations related to morphology suggest that multi-parameter evaluation protocols ought to replace single-parameter monitoring.
2. Morphological assessment should be more heavily weighted in treatment monitoring protocols, as it could arguably act as an early predictor of overall treatment success due to its pivotal role in posttreatment improvements.
3. The differential patterns of loading and its correlation in younger vs. older patients suggest that age-stratified treatment approaches may be warranted with an increased emphasis on early intervention to achieve optimal outcomes.
4. Consistency in group results should not be expected, as suggested by the reduction of total variance explained (71% → 69%) indicating that treatment effect is more heterogeneous and individualized.
5. Anti-inflammatory strategies should be integrated into treatment protocols, given the observed attenuation of leukocyte-defect associations posttreatment.

These findings extend beyond traditional semen analysis interpretation by suggesting how successful treatments may be associated with reshaping of parameter relationships, with patterns consistent with more integrated sperm function — observations that, if confirmed prospectively, could meaningfully inform clinical monitoring strategies.

Several limitations of this study warrant explicit discussion. First, the retrospective design precludes control over unmeasured confounders. Lifestyle factors with established associations with semen quality — including smoking, alcohol consumption, body mass index, physical activity, and dietary patterns — were not systematically recorded and could not be adjusted for in the analysis. Second, treatment modalities were heterogeneous and individualized, and no stratification by treatment type was implemented in the present analysis. The observed longitudinal associations in semen parameter structure therefore cannot be attributed to any specific intervention, and treatment-specific subgroup analyses were not feasible. Third, the absence of a concurrent control group — that is, untreated infertile men followed over an equivalent time period — means that observed posttreatment changes may partly reflect natural biological variation, regression to the mean, or the effect of repeated semen analysis rather than treatment-related effects exclusively. Fourth, the age-stratified patterns reported here are based on continuous age loadings in PCA rather than formal subgroup analyses with defined age categories, and should be interpreted as exploratory observations rather than definitive age-stratified conclusions. Fifth, the moderate KMO values and differing variable sets between PCA models impose constraints on the strength of structural comparisons across timepoints, as discussed in the Methods section. Collectively, these limitations underscore the need for prospective, controlled, multi-center studies with standardized treatment protocols, systematic lifestyle data collection, defined age strata, and clinical follow-up to confirm and extend the present findings.

The major limitation of this study is the lack of clinical fertility outcomes such as natural conception rates, fertilization in ART and live birth rates. Therefore, at this stage, the clinical translational relevance of the identified semen parameter remodeling is still uncovered. Although we witness remarkable biologically relevant shifts at the biomarker level with regards to strengthening of morphology–motility correlations, and emergence of an integrated PCA component posttreatment, from our data we cannot determine that these multivariate changes have been associated with any changes in clinical reproductive outcome. Thus, the current results must be viewed as hypothesis-generating observations that pave the way for, but do not supplant, prospective studies of trending PCA-derived parameters in relation to clinical endpoints. Future studies accounting for pregnancy rates and outcomes of assisted reproduction would be necessary to assess if this systems-level reorganization indeed possesses true predictive utility over fertility outcome.

## Conclusions

This study demonstrates that male infertility treatment was associated with systematic reorganization of semen parameter relationships, rather than isolated improvements, a finding supported by convergent evidence from both correlation matrices and principal component analyses. The persistence of strong interrelationships among sperm concentration, total sperm number, and progressive motility (rs ≥ 0.59, p < 0.001) suggests that these remain the core fertility indicators before and after treatment. However, motility’s reduced loading on PC1 (0.79 → 0.58) and its emergence as a significant contributor to PC2 (loading 0.59) suggest a redistribution of variance observed following treatment — a pattern consistent with a shift from purely quantitative gains toward more integrated sperm function, though this interpretation remains hypothesis-generating given the study design. A particularly striking finding was the strengthening of morphology-related correlations, which increased 32–76% across key parameters posttreatment. PCA supported this by revealing a transition of PC2 from a defect-dominated component toward what may be described as a ‘functional integration’ axis coupling morphology with motility. These observations align with established evidence that morphologically normal sperm exhibit superior motility and fertilizing potential, and raise the hypothesis that morphology may serve as an early, sensitive marker of treatment response — a proposition that future prospective studies should examine directly, providing clinicians with a more informative outcome metric than concentration alone.

Age-dependent response patterns were also revealed. Younger men showed stronger morphology–motility integration, while older men exhibited persistent challenges with liquification and structural defects, consistent with known age-related declines in sperm quality. These findings support age-stratified treatment counseling and underscore the importance of early intervention for optimizing outcomes. Finally, the attenuation of leukocyte-defect associations and the exclusion of WBC from posttreatment PCA suggest resolution of inflammatory contributions to sperm abnormalities. This is consistent with the potential importance of integrating anti-inflammatory and antioxidant strategies into treatment protocols, though the mechanistic role of such interventions requires prospective confirmation. Collectively, these results advocate for a paradigm shift in male fertility assessment. Single-parameter monitoring may be insufficient; multivariate approaches could more comprehensively capture longitudinal changes in semen parameter networks observed between pre and posttreatment assessments. Morphology, in particular, may merit greater weight in treatment monitoring as a potential biomarker of treatment response — though its predictive value for downstream fertility potential requires confirmation in studies with clinical outcome data. As detailed in the limitations discussion above, the retrospective design, treatment heterogeneity, and absence of a control group mean that the observed multivariate patterns should be interpreted as longitudinal associations rather than effects attributable to specific interventions. Most critically, the absence of clinical outcome data — including conception rates and assisted reproduction success — means that the observed biomarker-level reorganization cannot yet be linked to improved patient outcomes. Future prospective studies incorporating clinical endpoints are essential to determine whether the systems-level parameter reorganization documented here translates into meaningful gains in fertility success.

## Supplementary Information

Supplementary Material 1: Table 1. Characteristics of patients before and after treatment. Supplementary Fig.1 Treatment Protocol. Flowchart summarizing patient individualized treatment allocation, timing between pre- and posttreatment semen analyses (median: 163 days), and standardized abstinence periods (2–7 days, median: 3 days).

## Data Availability

No datasets were generated or analysed during the current study.
