# Supplementary material for: Assessing the treatment of male infertility through principal component analysis: a retrospective study
Source: Basic Clin Androl. 2026 Jul 7;36:19. doi: 10.1186/s12610-026-00317-1 (PMC13340328; doi:10.1186/s12610-026-00317-1)
Supplement: Supplementary file 1 [file 12610_2026_317_MOESM1_ESM.odt]

Supplementary Information 

Table 1. Characteristics of patients before and after treatment

|  | (Mean ± SD) |
| --- | --- |
| Variable | Pretreatment | Posttreatment |
| Age (years) | 33.4 ± 6.3 | 34 ± 6.4 |
| Liquification time (minutes) | 43 ± 37 | 41.8 ± 31.8 |
| Abstinence days | 3.8 ± 1.6 | 3.7 ± 1.3 |
| WBC (×10⁶/mL) | 0.43 ± 0.64 | 0.39 ± 0.58 |
| Volume (mL) | 3.0 ± 1.5 | 3.0 ± 1.6 |
| Sperm concentration (×10⁶/mL) | 35.95 ± 32.38 | 36.86 ± 32.74 |
| Total sperm number (×10⁶/ejaculate) | 109.5 ± 119.0 | 113.4 ± 129.9 |
| Progressive motility | 0.33 ± 0.22 | 0.30 ± 0.23 |
| Normal morphology | 0.03 ± 0.06 | 0.03 ± 0.07 |

Note: Values are presented as mean ± SD. Statistical analysis: Descriptive statistics only; no inferential            statistical test was applied. WBC = white blood cells; SD = standard deviation

Supplementary Fig.1 Treatment Protocol
 Flowchart summarizing patient individualized treatment allocation, timing between pre- and posttreatment semen analyses (median: 163 days), and standardized abstinence periods (2–7 days, median: 3 days).
